# Temporal Cues Influence Space Estimations in Visually Impaired Individuals

**DOI:** 10.1016/j.isci.2018.07.003

**Published:** 2018-08-01

**Authors:** Monica Gori, Maria Bianca Amadeo, Claudio Campus

**Affiliations:** 1 U-VIP Unit for Visually Impaired People, Fondazione Istituto Italiano di Tecnologia, Via E. Melen, 83, Genova 16152, Italy; 2 Department of Informatics, Bioengineering, Robotics and Systems Engineering, Università degli Studi di Genova, Via all'Opera Pia, 13, Genova 16145, Italy

**Keywords:** Disability, Neuroscience, Cognitive Neuroscience

## Abstract

Many works have highlighted enhanced auditory processing in blind individuals, suggesting that they compensate for lack of vision with greater sensitivity of the other senses. Few years ago, we demonstrated severely impaired auditory precision in congenitally blind individuals performing an auditory spatial metric task: their thresholds for bisecting three consecutive spatially distributed sounds were seriously compromised, ranging from three times typical thresholds to total randomness. Here, we show that the deficit disappears if blind individuals are presented with coherent temporal and spatial cues. More interestingly, when the audio information is presented in conflict for space and time, sighted individuals are unaffected by the perturbation, whereas blind individuals are strongly attracted by the temporal cue. These results highlight that temporal cues influence space estimations in blind participants, suggesting for the first time that blind individuals use temporal information to infer spatial environmental coordinates.

## Highlights

• Blind individuals are not able to perform auditory spatial metric tasks
• Their deficit disappears when coherent temporal and spatial cues are presented
• In some cases, blind people use temporal cues to infer spatial coordinates

## Graphical Abstract

**Figure:**
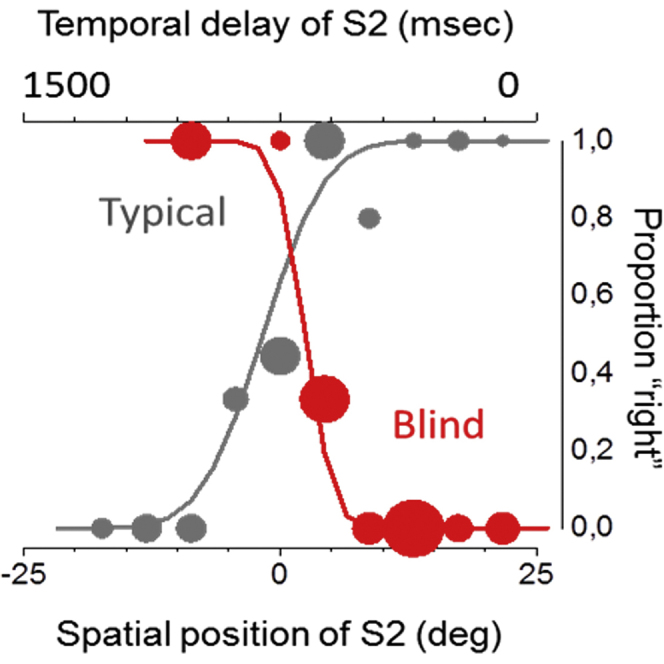

## Introduction

The ability to build spatial coordinates combining neural signals from different sensory modalities is fundamental to allow coherent perception and interaction with the environment. Converging evidence from animal studies suggests that the development of multisensory interactions between vision and other senses depends on early perceptual experience. Although multisensory neurons are present in newborn monkeys, they acquire their spatially specific multimodal properties only during the first few months of life, in an experience-dependent fashion (Wallace and Stein, 2001). Given the superiority of vision over the other sensory systems for space representation, the visual modality might offer a spatial background for remapping other sensory information. Supporting this idea, the literature suggests that eye-centered coordinates are used to align neural representations of space for different sensory modalities in the brain (Pouget et al., 2002, King, 2009, Jay and Sparks, 1984, Cohen and Andersen, 2002). In this regard, the representation of auditory (external) space is dominated by visual experience in young children (Gori et al., 2012). Moreover, research in animals shows that auditory spatial maps of juvenile barn owls change after adaptation with prismatic spectacles that causes horizontal displacements of visual space (Knudsen, 1998). Adult owls show similar recalibrations only if exposed to prismatic visual displacements as juveniles. No recalibration occurs for adult owls without this early experience. All these findings clearly indicate that the lack of visual experience in blind infants might interfere with the development of a coherent spatial representation of the environment.

Blindness is a unique condition to investigate the role of the visual modality on the development of spatial representation. On the one side, when visual experience is not available the auditory modality seems to be a good substitute to interpret some spatial representations, with blind individuals showing enhanced auditory spatial skills compared with sighted individuals (e.g., King and Parsons, 1999, Roder et al., 1999, Gougoux et al., 2005, Lewald, 2002, Lessard et al., 1998, Zwiers et al., 2001). At the neurophysiological level, in blind individuals occipital areas deprived of the visual input start to be activated by auditory stimuli (e.g., Weeks et al., 2000, Poirier et al., 2005, Renier and De Volder, 2005, Striem-Amit and Amedi, 2014, Gougoux et al., 2005), and responses in these areas to auditory stimuli appear to be organized in a topographic manner (Voss and Zatorre, 2012, Collignon et al., 2009, Collignon et al., 2011, Collignon et al., 2013, Rauschecker, 1995). Changes within the auditory pathway in the absence of visual input have also been reported in blind individuals (e.g., Korte and Rauschecker, 1993, Elbert et al., 2002). On the other side, the lack of visual experience affects the development of other auditory spatial skills. For example, a few years ago we reported that blind individuals show strong deficits in the performance of the audio space bisection task (Gori et al., 2014, Vercillo et al., 2016). In the space bisection task, the individual has to listen to three sounds delivered from three different spatial positions and evaluate whether the second one in between the other two is closer in space to the first or to the last one. The cortical areas associated with the processing of this stimulus have been recently investigated by our group, suggesting different patterns of activation among sighted and blind individuals. Sighted individuals show an early occipital ERP response (50–90 ms) for this task, which mimics the C1 ERP component usually elicited by visual tasks (Campus et al., 2017).

Why are blind individuals, who have higher auditory skills compared with sighted individuals for many spatial tasks, not able to perform the space bisection task? Although this task requires complex attentional and memory skills, it is hard for blind individuals only in the spatial domain. Indeed, when they have to bisect stimuli in the temporal domain, their performance is as good as those of sighted individuals, as well as their cortical activations (Campus et al., 2017).

Almost 100 years ago, Piaget and Inhelder (1962) stated that the temporal metric is strictly related to spatial metric development. “Space is a still of time, while time is space in motion” (Piaget, 1927, p.2). What Jean Piaget did not discuss is the role of different sensory modalities on this link. As we have discussed earlier, the visual experience is important for the development of spatial metric representations, such as for bisecting sounds. Starting from Piaget's idea, one might hypothesize that, when vision is not available, such as in blindness, temporal representation of events could be used to set spatial representations. If this is the case, we expect space representations of blind individuals to be strongly influenced by the temporal representation of events.

Here we tested and verified this hypothesis. Sighted and blind individuals performed various space bisection tasks, in which spatial and temporal coherent and conflicting information were presented. In blind individuals, the spatial bisection deficit disappeared when coherent temporal and spatial cues were presented (e.g., short space associated with short time) and increased when conflicting spatial and temporal information was presented (e.g., short space associated with a long time). Our results suggest that blind individuals are strongly attracted by temporal cues to infer metric spatial information. These findings support the idea that temporal representation is crucial for the development of spatial metric representations, and the visual experience is fundamental for this mediation to occur.

## Results

**Figure 1:**
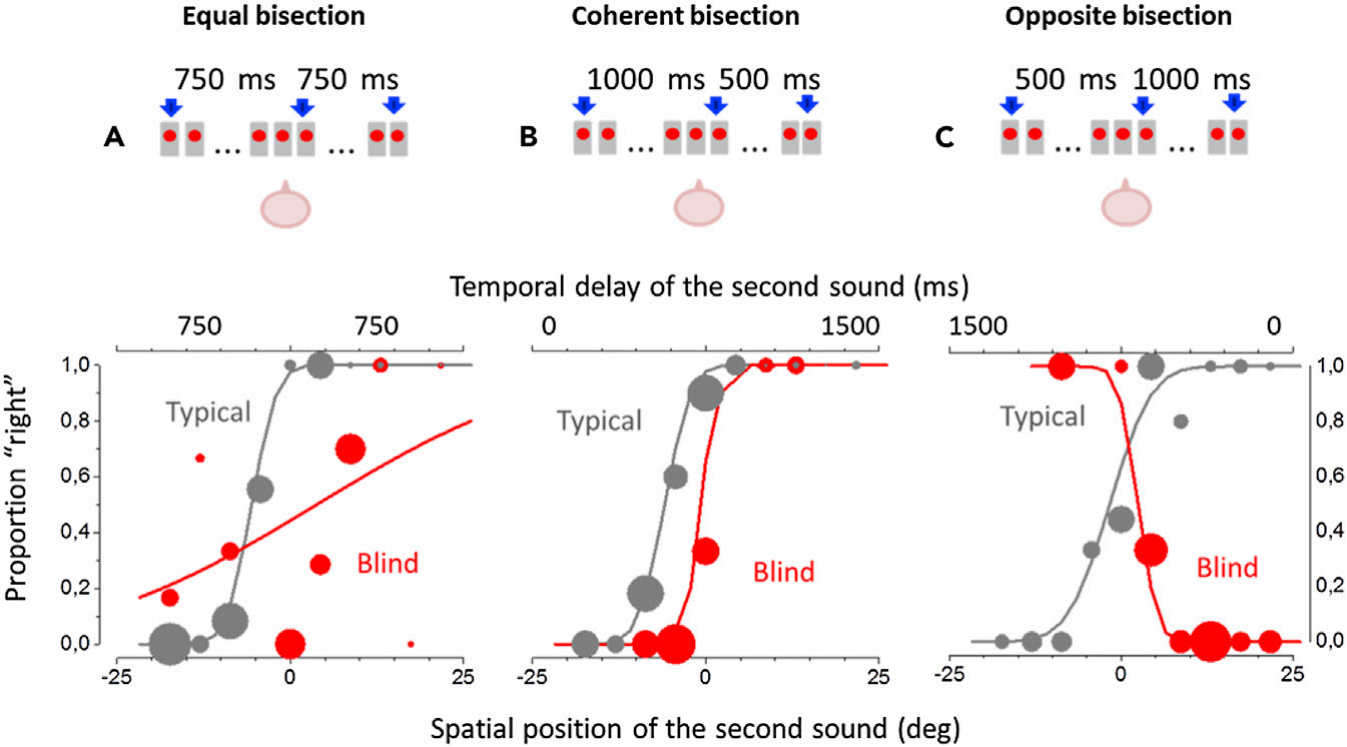
Auditory Spatial Bisection Tasks Results of the three conditions for a typical blind participant (red symbols) and a typical sighted control (gray symbols). Subjects sat in front of an array of 23 speakers, illustrated by the sketches above the graphs. (A) Equal spatial bisection. Top: the time interval between the first and the second sound (750 ms) was equal to the time interval between the second and the third sound. Bottom: proportion of trials judged “closer to the right sound source,” plotted against the speaker position for the second sound. The size of the dots is proportional to the number of trials at that position. Both sets of data are fitted with the Gaussian error function. (B) Coherent spatial bisection. Top: spatial distances and temporal intervals between the three sounds were directly proportional (e.g., long spatial distance and long temporal interval). Bottom: same as for (A). (C) Opposite spatial bisection. Top: spatial distances and temporal intervals between the three sounds were inversely proportional (e.g., long spatial distance and short temporal interval). Bottom: same as for (A) and (B).

A total of 17 blind adults (see Table S1 for details) and 17 age-matched controls performed four auditory bisection tasks: three spatial bisection tasks and one temporal bisection task as a control experiment. In the three spatial bisection tasks (*equal time*, *coherent time*, and *opposite time*), aimed to measure thresholds for spatial bisection, three consecutive sounds were presented (see Figure 1, upper panels), and subjects judged whether the second sound was spatially closer to the first (displayed at −25°, left of center) or to the third (+25°, right of center) sound. To evaluate the role of temporal cues on spatial bisection performance of blind individuals, the temporal interval between the three sounds was manipulated in the three spatial tasks. In the *equal time* spatial bisection task, the three sounds were played with the same delay between the first and the second sound and between the second and the third sound (750 ms; as in original work, Gori et al., 2014). In this case, only spatial cues were relevant to compute the task as the temporal delay between the three sounds was the same (Figure 1A top). In the *coherent time* spatial bisection task, spatial distances and temporal intervals between the three sounds were directly proportional: a longer spatial distance between the first and the second sound was associated with a longer temporal delay between the two sounds, and the reverse for shorter distances (Figure 1B top). In the *opposite time* spatial bisection task, spatial distances and temporal intervals between the three sounds were inversely proportional: a longer spatial distance between the first and the second sound was associated with a shorter temporal delay between the two sounds, and the reverse for shorter distances (Figure 1C top). In the temporal bisection task performed as a control, subjects had to listen to three subsequent sounds produced by the same speaker and report if the second sound was closer to the first one or to the last one by using temporal cues. For more information see Transparent Methods in Supplemental Information.

Figure 1 (lower panels) plots the proportion of answer “second sound closer to the third sound” as a function of the position of the second sound, for one typical blind subject (in red) and one age-matched typical, sighted control (in gray). The size of the dots is proportional to the number of trials at each position. Figure 1A reports the results for the *equal* bisection condition, Figure 1B for the *coherent* bisection condition, and Figure 1C for the *opposite* bisection condition. In the *equal* bisection condition (Figure 1A), the sighted individual shows the typical psychometric function. Contrarily, the blind subject shows almost random responses with no psychometric function, reflecting strong impairment in this task (in agreement with previous findings, Gori et al., 2014). As regards the *coherent* bisection task (Figure 1B), the results are quite different: here the psychometric function for the blind individual is present and similar to that of the sighted participant, meaning similar precision. This result suggests that a temporal cue can be used by blind individuals to improve their performance in the space bisection task. In the *opposite* spatial bisection task (Figure 1C), the response of the sighted subject is identical to the response in the other two conditions. Differently, the blind individual shows a well-shaped psychometric function but in the opposite direction than expected (in gray). The performance of the blind individual reveals a strong temporal dominance for the space bisection task under this condition, suggesting that in this blind subject, whereas not in the sighted one, the temporal cue is attracting the spatial auditory response.

**Figure 2:**
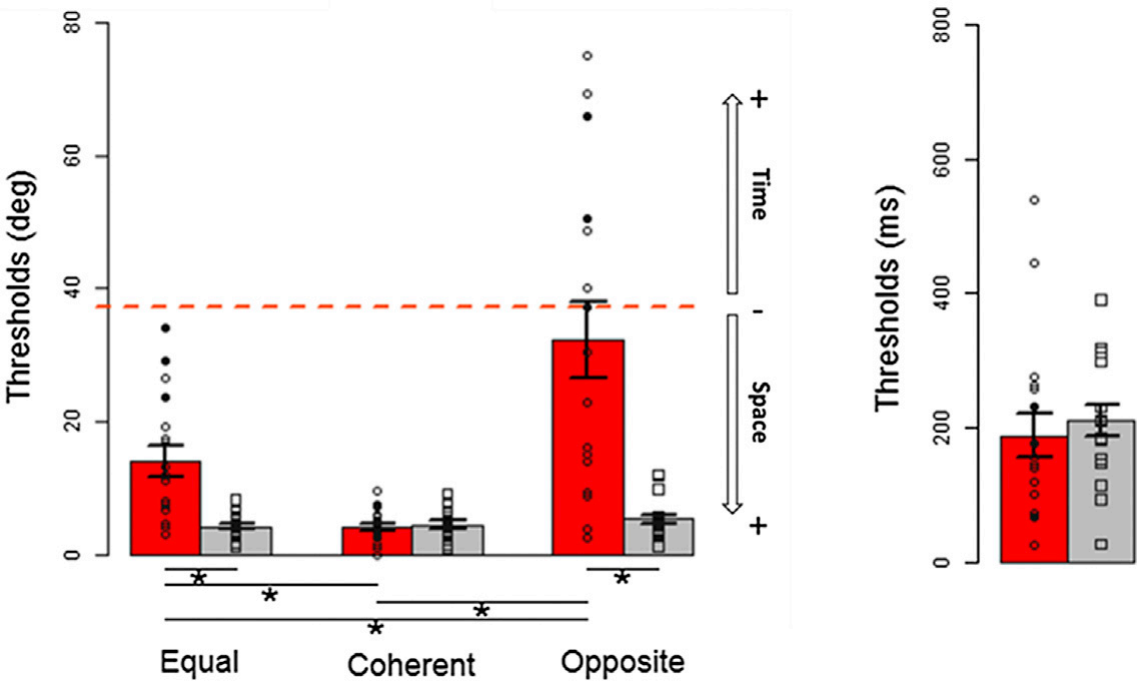
Group Performance in Auditory Bisection Tasks Average thresholds (±SEM) of the three spatial bisection tasks (left panel) and the temporal bisection task (right panel) for blind (red; see also Table S1) and sighted (gray) participants. White dots (early blind), black dots (late blind), and squares (sighted) represent individual data; dots above the red line indicate subjects with inverted psychometric function. *p < 0.01 after Bonferroni correction.

Figure 2 shows the results for all subjects involved in the study. Averages and individual data for the three spatial bisection tasks and for the temporal bisection task are reported for blind (in red) and sighted (in gray) individuals. Dots represent individual data, and those above the red dashed line indicate subjects with inverted psychometric function. For sighted individuals (in gray) the manipulation of the temporal cue during space bisection (Figure 2 left panel) does not affect the response (i.e., the gray bars are at the same level for the three spatial conditions), whereas it strongly influences the response of blind participants. As previously suggested by the psychometric functions reported in Figure 1, the average threshold of blind participants (red bar) is higher than that of sighted participants for the *equal* space bisection (in agreement with previous findings, Gori et al., 2014), but average thresholds become similar between the groups for the *coherent* space bisection, suggesting that blind individuals benefit from the temporal cue during spatial judgments. This interaction seems to occur under threshold. The smallest difference between the temporal delays of S2 was of 65 ms for each speaker, whereas the temporal threshold obtained from the temporal bisection task was of 200 ms. Considering the coherent condition in Figure 2, we can observe that the spatial threshold is on average 3.5°, meaning less than two speakers. This spatial threshold corresponds to a temporal delay of about 130 ms, which is lower than the temporal threshold obtained in temporal bisection, suggesting subthreshold interaction between space and time. Since an interaction between space and time has been reported for the detection of motion stimuli (Burr et al., 1986) and subthreshold facilitation is an evidence of functional interaction at early levels of sensory processing (Gori et al., 2011), we can speculate that the interaction between space and time we observed occurs at early sensory level. Importantly, the thresholds of blind participants increase in the *opposite* bisection task, with some participants inverting the psychometric function (i.e., those with thresholds above the red dashed line). This result implies a reduction of precision in the conflict condition. The two-way ANOVA with spatial thresholds as dependent variable claims a significant interaction (F_2,64_ = 17.72, p = 7*10^−7^, generalized eta squared = 0. 23) between group (sighted, blind) and task (*equal time*, *coherent time*, *opposite time*). From follow-up one-way ANOVAs, significant differences among tasks (*equal time*, *coherent time*, *opposite time*) emerge for the blind group (F_2,32_ = 19.34, p = 3*10^−6^, generalized eta squared = 0. 4) but not for the sighted group (F_2,32_ = 1.5, p = 0.2, generalized eta squared = 0. 03). *Post- hoc* t tests reveal that the performance of blind individuals is statistically more impaired in the *opposite time* bisection task compared with their own performance in the *equal time* (t_16_ = 3.7, p = 0.006) and *coherent time* (t_16_ = −4.86, p = 0.0005) condition. However, their performance significantly improves from the *equal time* to the *coherent time* condition (t_16_ = −4.21, p = 0.002). In addition, spatial thresholds of blind participants are significantly higher than those of sighted participants in the *equal* (blind vs. sighted: t_17.1_ = 4.18, p = 0.002) and *opposite time* (blind vs. sighted: t_16.6_ = 4.69, p = 0.0007) conditions. The role of time cues in inferring spatial metric is also evident by low thresholds and no statistical differences between groups in the *coherent time* spatial bisection task (blind vs. sighted: t_32_ = −0.45, p = 1). In agreement with previous results (Gori et al., 2012, Vercillo et al., 2016), all participants were able to perform the temporal bisection task and similar precision is observed between sighted and blind groups (Figure 2 right panel; F_1,32_ = 0.29, p = 0.6, generalized eta squared = 0.009).

**Figure 3:**
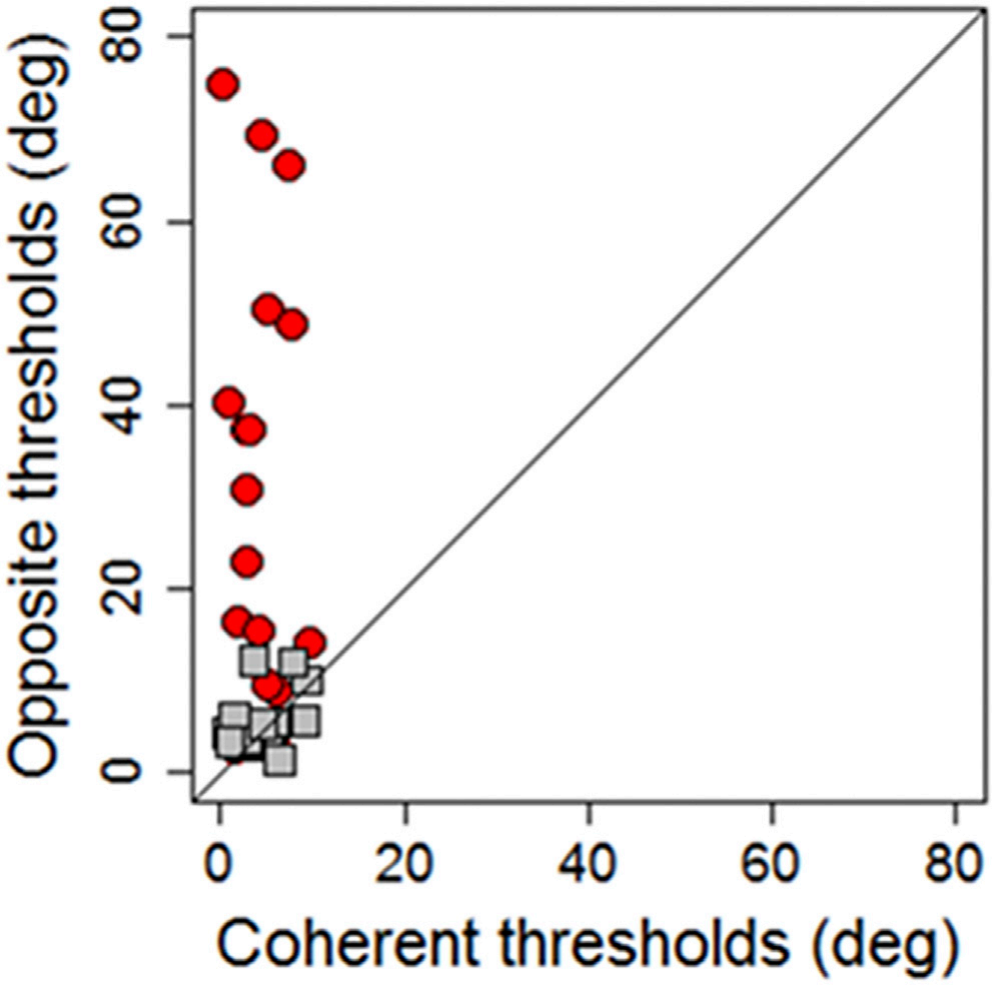
Relationship between Coherent and Opposite Spatial Bisection Tasks Individual data, plotting opposite thresholds against coherent thresholds (calculated from the width of individual psychometric functions). Red and gray dots represent blind and sighted individuals, respectively.

In Figure 3, individual thresholds in the *coherent time* spatial bisection task is plotted against individual thresholds in the *opposite time* spatial bisection task for the sighted (in gray) and blind (in red) groups. Sighted participants show similar performances for both tasks, with all the individual data lying in the equality line, whereas blind participants display discrepancies between thresholds in the two tasks. In this latter group, all dots lie above the equality line, suggesting lower performance for the *opposite time* than the *coherent time* task.

## Discussion

Here we studied whether time attracts space for visually impaired individuals. In particular, we hypothesized that, in blind individuals, for whom the visual input is missing, temporal cues could be used to determine the spatial relationships of events. Blind and sighted subjects were tested with an audio task in which conflicting and not-conflicting spatial and temporal information were delivered. As predicted, we observed a strong attraction toward temporal cues during space bisection in blind but not in sighted individuals. In blind participants, the spatial bisection deficit (previously reported by Gori et al., 2014) disappears when coherent temporal and spatial cues are presented (e.g., short space associated with short time) and increases for conflicting spatial and temporal stimuli (e.g., short space associated with long time). These results provide two important points of discussion. The first one is that temporal and spatial representations are strictly linked: in blind individuals, the modification of temporal cues alters space bisection performance. The second one is that our visual experience seems to be crucial for the development of independent spatial and temporal representations: temporal attraction of space is evident only for blind and not for sighted individuals, who can dissociate the two cues without any problem.

The space bisection is a particular task in many aspects. Its visual static version has been widely used to study the visual neglect (e.g., Bultitude and Aimola Davies, 2006). It requires spatial representations in Euclidian coordinates and strong spatial skills in terms of memory and attention, and it taxes a sophisticated and well-calibrated spatial auditory map. Yet, what we think is special about the space bisection is that it naturally combines spatial with temporal representations. Both spatial and temporal intervals are determined by the first and the third stimuli, and the spatial and temporal coordinates of the second stimulus can be independently modulated with respect to the other two stimuli. In Gori et al. (2014), the spatial and temporal cues were conflicting (as in the equal condition tested here): the same temporal interval was applied for different spatial distances and, as a result, blind individuals reported random responses. By providing coherent spatial-temporal cues (i.e., in the coherent condition tested here), the deficit disappears and blind people perform the task similarly to sighted participants. Is this temporal attraction of space a general principle of space representation in blindness, or is it specific for the space bisection task? Although spatiotemporal interactions in blind individuals have also been observed in other studies (e.g., Roder et al., 2004), further research will be necessary to understand if this kind of interaction represents a general principle of the blind brain. A possible way to answer this question could be applying a similar approach to other tasks (such as the minimum audible angle), for which enhanced skills have been reported in blind individuals.

How space and time are represented in our brain and how different sensory modalities shape the development of these representations are still an open issue. Occipital areas are activated by auditory stimuli when the visual input is not available (e.g., Weeks et al., 2000, Poirier et al., 2005, Renier and De Volder, 2005, Striem-Amit and Amedi, 2014, Gougoux et al., 2005), and auditory responses in these areas are represented in a topographic manner (Recanzone, 1998, Zwiers et al., 2003, Gori et al., 2012, Burr and Gori, 2011). Our results suggest that in some cases the brain may use temporal cues to infer spatial coordinates of the environment. A possible speculation is that it assumes the constant velocity of the stimuli and consequently uses temporal maps to solve spatial metric analysis. This hypothesis would explain why blind individuals are strongly enhanced when spatiotemporal coherent cues are presented and invert the psychometric function when facing conflicting spatiotemporal information: they follow the virtual position of the second sound suggested by its temporal delay, using temporal cues to make specific audio spatial metric estimations.

Two main theories address how the concepts of space and time are linked in the human mind: the Metaphor Theory (MT, Lakoff and Johnson, 1999) and the Theory of Magnitude (ATOM; Walsh, 2003). The MT theory states that space unilaterally affects time, whereas the ATOM states that space and time, together with numbers, are represented in the brain by a common magnitude system and are thus symmetrically interrelated (Bueti and Walsh, 2009, Burr et al., 2010, Lambrechts et al., 2013). Different behavioral (e.g., Bueti and Walsh, 2009, Dormal et al., 2008) and neuroimaging works (e.g., Fias et al., 2003, Pinel et al., 2004, Dormal and Pesenti, 2009) agree with the ATOM theory, highlighting interferences between the two domains and the activation of overlapping areas in the parietal lobe during magnitude processing. By showing a temporal influence on spatial representations in blind individuals, our findings certainly sustain a Theory of Magnitude (Walsh, 2003). “Space is a still of time, while time is space in motion” (Piaget, 1927, p.2). This statement reported by Piaget almost 100 years ago also suggests that the development of our spatial representation is strictly related to our temporal representation of the environment. Our work confirms this idea, adding another evidence. The development of independent spatial and temporal representations for the space bisection task is mediated by the visual input. From our data, a possible speculation is that the temporal sequence of events is at the base of the development of spatial metric relationship understanding. Since the visual experience is important for the development of complex spatial representations, when vision is not available we might speculate that independent temporal and spatial maps cannot develop. As a result, in blind individuals, the temporal cues are used to determine the spatial relationship of events. Thus, our findings support the idea that, in auditory tasks, temporal metric representations are mediators for the development of spatial metric representations and the visual experience is crucial for this mediation to occur.

Previous works showed that the spatial reference frames of blind individuals are fundamentally different from those of sighted individuals (Pasqualotto et al., 2013). In agreement with this idea, we have recently shown that blind individuals enhance their skills in the spatial bisection task when they can use their body as a reference (Vercillo et al., 2018). A possible explanation might be that, when blind individuals have to face complex spatial representations they are not able to solve, they rely on alternative cues. One cue could be the body as a reference, the other one could be time. A few years ago, we proposed the theory of cross-sensory calibration (Gori, 2015). It states that, during development, sensory channels communicate with each other and can be used to calibrate the sensory signals. The most robust, accurate sensory information (i.e., vision for object orientation, Gori et al., 2010) can be used to calibrate the other sensory signals (i.e., the haptic one). The present study sheds light on new possible interactions during development, not only among sensory modalities but also among spatial and temporal domains. Besides the obvious theoretical relevance in demonstrating the importance of cross-sensory interactions for normal development (Burr and Gori, 2011), our results could have repercussions for rehabilitation. Blind people rely strongly on auditory information to orient them in the environment. Sturdy spatial maps are clearly of paramount importance, and their development in the absence of visual information has to be understood and recovered if impaired. New techniques could be realized whereby spatial and temporal cues could be simultaneously manipulated to convey richer information. Moreover, we open new opportunities for the development of sensory substitution devices and rehabilitation technologies whereby temporal cues can be used to recalibrate spatial representation in blind individuals.

## Methods

All methods can be found in the accompanying Transparent Methods supplemental file.

## Supplemental Information

Document S1. Transparent Methods and Table S1

## References

[bib1] Bueti D., Walsh V. (2009). The parietal cortex and the representation of time, space, number and other magnitudes. Philos. Trans. R. Soc. Lond. B Biol. Sci..

[bib2] Bultitude J.H., Aimola Davies A.M. (2006). Putting attention on the line: investigating the activation-orientation hypothesis of pseudoneglect. Neuropsychologia.

[bib3] Burr D., Gori M., Murray M.M., Wallace M.T. (2011). Multisensory integration develops late in humans. The Neural Bases of Multisensory Processes.

[bib4] Burr D.C., Ross J., Binda P., Morrone M.C. (2010). Saccades compress space, time and number. Trends Cogn. Sci..

[bib5] Burr D.C., Ross J., Morrone M.C. (1986). Seeing objects in motion. Proc. R. Soc. Lond. B Biol. Sci..

[bib6] Campus C., Sandini G., Concetta Morrone M., Gori M. (2017). Spatial localization of sound elicits early responses from occipital visual cortex in humans. Sci. Rep..

[bib7] Cohen Y.E., Andersen R.A. (2002). A common reference frame for movement plans in the posterior parietal cortex. Nat. Rev. Neurosci..

[bib8] Collignon O., Champoux F., Voss P., Lepore F. (2011). Sensory rehabilitation in the plastic brain. Prog. Brain Res..

[bib9] Collignon O., Charbonneau G., Peters F., Nassim M., Lassonde M., Lepore F., Mottron L., Bertone A. (2013). Reduced multisensory facilitation in persons with autism. Cortex.

[bib10] Collignon O., Voss P., Lassonde M., Lepore F. (2009). Cross-modal plasticity for the spatial processing of sounds in visually deprived subjects. Exp. Brain Res..

[bib11] Dormal V., Andres M., Pesenti M. (2008). Dissociation of numerosity and duration processing in the left intraparietal sulcus: a transcranial magnetic stimulation study. Cortex.

[bib12] Dormal V., Pesenti M. (2009). Common and specific contributions of the intraparietal sulci to numerosity and length processing. Hum. Brain Mapp..

[bib13] Elbert T., Sterr A., Rockstroh B., Pantev C., Muller M.M., Taub E. (2002). Expansion of the tonotopic area in the auditory cortex of the blind. J. Neurosci..

[bib14] Fias W., Lammertyn J., Reynvoet B., Dupont P., Orban G.A. (2003). Parietal representation of symbolic and nonsymbolic magnitude. J. Cogn. Neurosci..

[bib15] Gori M. (2015). Multisensory integration and calibration in children and adults with and without sensory and motor disabilities. Multisens. Res..

[bib16] Gori M., Mazzilli G., Sandini G., Burr D. (2011). Cross-sensory facilitation reveals neural interactions between visual and tactile motion in humans. Front. Psychol..

[bib17] Gori M., Sandini G., Burr D. (2012). Development of visuo-auditory integration in space and time. Front. Integr. Neurosci..

[bib18] Gori M., Sandini G., Martinoli C., Burr D. (2010). Poor haptic orientation discrimination in nonsighted children may reflect disruption of cross-sensory calibration. Curr. Biol..

[bib19] Gori M., Sandini G., Martinoli C., Burr D.C. (2014). Impairment of auditory spatial localization in congenitally blind human subjects. Brain.

[bib20] Gougoux F., Zatorre R.J., Lassonde M., Voss P., Lepore F. (2005). A functional neuroimaging study of sound localization: visual cortex activity predicts performance in early-blind individuals. PLoS Biol..

[bib21] Jay M.F., Sparks D.L. (1984). Auditory receptive fields in primate superior colliculus shift with changes in eye position. Nature.

[bib22] King A.J. (2009). Visual influences on auditory spatial learning. Philos. Trans. R. Soc. Lond. B Biol. Sci..

[bib23] King A.J., Parsons C.H. (1999). Improved auditory spatial acuity in visually deprived ferrets. Eur. J. Neurosci..

[bib24] Knudsen E.I. (1998). Capacity for plasticity in the adult owl auditory system expanded by juvenile experience. Science.

[bib25] Korte M., Rauschecker J.P. (1993). Auditory spatial tuning of cortical neurons is sharpened in cats with early blindness. J. Neurophysiol..

[bib26] Lakoff G., Johnson M. (1999). Philosophy in the Fiesh: The Embodied Mind and its Challenge to Western Thought.

[bib27] Lambrechts A., Walsh V., van Wassenhove V. (2013). Evidence accumulation in the magnitude system. PLoS One.

[bib28] Lessard N., Pare M., Lepore F., Lassonde M. (1998). Early-blind human subjects localize sound sources better than sighted subjects. Nature.

[bib29] Lewald J. (2002). Vertical sound localization in blind humans. Neuropsychologia.

[bib30] Pasqualotto A., Spiller M.J., Jansari A.S., Proulx M.J. (2013). Visual experience facilitates allocentric spatial representation. Behav. Brain Res..

[bib31] Piaget J. (1927). The Child’s Conception of Time.

[bib32] Piaget J., Inhelder B. (1962). The Psychology of the Child.

[bib33] Pinel P., Piazza M., Le Bihan D., Dehaene S. (2004). Distributed and overlapping cerebral representations of number, size, and luminance during comparative judgments. Neuron.

[bib34] Poirier C., Collignon O., Devolder A.G., Renier L., Vanlierde A., Tranduy D., Scheiber C. (2005). Specific activation of the V5 brain area by auditory motion processing: an fMRI study. Brain Res. Cogn. Brain Res..

[bib35] Pouget A., Deneve S., Duhamel J.R. (2002). A computational perspective on the neural basis of multisensory spatial representations. Nat. Rev. Neurosci..

[bib36] Rauschecker J.P. (1995). Developmental plasticity and memory. Behav. Brain Res..

[bib37] Recanzone G.H. (1998). Rapidly induced auditory plasticity: the ventriloquism aftereffect. Proc. Natl. Acad. Sci. USA.

[bib38] Renier L., De Volder A.G. (2005). Cognitive and brain mechanisms in sensory substitution of vision: a contribution to the study of human perception. J. Integr. Neurosci..

[bib39] Roder B., Rosler F., Spence C. (2004). Early vision impairs tactile perception in the blind. Curr. Biol..

[bib40] Roder B., Teder-Salejarvi W., Sterr A., Rosler F., Hillyard S.A., Neville H.J. (1999). Improved auditory spatial tuning in blind humans. Nature.

[bib41] Striem-Amit E., Amedi A. (2014). Visual cortex extrastriate body-selective area activation in congenitally blind people “seeing” by using sounds. Curr. Biol..

[bib42] Vercillo T., Burr D., Gori M. (2016). Early visual deprivation severely compromises the auditory sense of space in congenitally blind children. Dev. Psychol..

[bib43] Vercillo T., Tonelli A., Gori M. (2018). Early visual deprivation prompts the use of body-centered frames of reference for auditory localization. Cognition.

[bib44] Voss P., Zatorre R.J. (2012). Organization and reorganization of sensory-deprived cortex. Curr. Biol..

[bib45] Wallace M.T., Stein B.E. (2001). Sensory and multisensory responses in the newborn monkey superior colliculus. J. Neurosci..

[bib46] Walsh V. (2003). A theory of magnitude: common cortical metrics of time, space and quantity. Trends Cogn. Sci..

[bib47] Weeks R., Horwitz B., Aziz-Sultan A., Tian B., Wessinger C.M., Cohen L.G., Hallett M., Rauschecker J.P. (2000). A positron emission tomographic study of auditory localization in the congenitally blind. J. Neurosci..

[bib48] Zwiers M.P., Van Opstal A.J., Cruysberg J.R. (2001). A spatial hearing deficit in early-blind humans. J. Neurosci..

[bib49] Zwiers M.P., Van Opstal A.J., Paige G.D. (2003). Plasticity in human sound localization induced by compressed spatial vision. Nat. Neurosci..

