# Supplementary material for: Temporal Cues Influence Space Estimations in Visually Impaired Individuals
Source: iScience. 2018 Aug 1;6:319–26. doi: 10.1016/j.isci.2018.07.003 (PMC6137691; doi:10.1016/j.isci.2018.07.003)
Supplement: Document S1. Transparent Methods and Table S1 [file mmc1.pdf]

ISCI, Volume *6*

Supplemental Information

Temporal Cues Influence Space Estimations in Visually Impaired Individuals

Monica Gori, Maria Bianca Amadeo, and Claudio Campus

Supplemental Information

PARTICIPANT	AGE	GENDER	PATHOLOGY	BLINDNESS ONSET
S1	52	M	Retinopathy of Prematurity	Birth
S2	77	F	Retinis Pigmentosa	Birth
S3	62	F	Atrophy of the eyeball	Birth
S4	25	M	Leber amaurosis	Birth
S5	52	F	Retinis Pigmentosa	Birth
S6	58	M	Uveitis	Birth
S7	59	M	Glaucoma	Birth
S8	42	F	Glaucoma	Birth
S9	28	F	Retinopathy of Prematurity	Birth
S10	27	F	Retinopathy of Prematurity	Birth
S11	24	F	Glaucoma	Birth
S12	27	F	Microphthalmia	Birth
S13	29	F	Retinis Pigmentosa	Birth
S14	29	M	Glaucoma	Birth
S15	65	M	Retinis Pigmentosa	38
S16	58	M	Glaucoma	20
S17	67	M	Retinal detachment	51

Table S1. Clinical details of the blind group (N= 17), Related to Figure 2.
The table shows chronological age at testing, gender, pathology and age of blindness onset for each participant.

Transparent Methods

Participants

A group of 17 blind participants (mean age: 45.9±17.9 yo; F=9) and 17 age and gender-matched sighted participants (36.5±13.5 yo; F=9; t_{32}= 1.74, p=0.1) took part in the study (see Table S1 for details). All participants reported no history of neurological or cognitive deficits. The research protocol was approved by the ethics committee of the local health service (Comitato Etico, ASL3 Genovese, Italy) and conducted in line with the Declaration of Helsinki. Written informed consent was obtained prior to testing.

Stimuli and procedure

Participants were sitting blindfolded in front of the center of an array of 23 speakers placed at a distance of 180 cm and spanning ±25° of visual angle (with 0° representing the central speaker, negative values on the left, and positive values on the right; Fig. 1, upper panels). They performed three spatial bisection tasks, and one temporal bisection task as a control. The order of spatial and temporal blocks was counterbalanced across subjects. In each task, subjects listened to a sequence of three

consecutive sounds (500 Hz, 75 ms duration, 60 dB Sound Pressure Level (SPL)) for a trial duration of 1500ms.

In spatial bisection tasks, participants judged verbally whether the second sound (S2) was spatially closer to first sound (S1; −25°, 0ms) or to third sound (S3; +25°, 1500ms). S2 could occur randomly at an intermediate position from -25° to +25° in space, determined by the QUEST adaptive algorithm (Watson & Pelli, 1983). In order to evaluate the role of temporal cues in space performance, temporal intervals between the three sounds were manipulated to originate three different spatial bisection tasks (Fig. 1, upper panels from left to right): *equal time*, *coherent time* and *opposite time* spatial bisection tasks, with time intervals which could be equal, coherent or opposite with respect to space distances respectively. In the *equal time* spatial bisection, S2 was always delivered 750ms after S1, which corresponded to the middle time of the temporal sequence between S1-S3. To correctly compute this task participants had to rely exclusively on spatial features since the three sounds were played with the same temporal delay between S1-S2, and between S2-S3 (as in original work, Gori, Sandini, Martinoli, & Burr, 2014), making temporal aspects uninformative. Among spatial bisection tasks, the *equal time* one was always performed as the first one, with the order of the other two tasks randomly varying across participants. In the *coherent time* spatial bisection task, spatial distances between S1-S2 and S2-S3 were directly proportional to temporal intervals between the three sounds (e.g. a shorter spatial distance between S1-S2 was associated with a shorter temporal delay between the two sounds). The exact temporal delay associated with each spatial position of S2 is reported in the upper horizontal axis of the central psychometric function in Figure 1. Considering that the total trial duration was 1500ms and the number of speakers was 23, when S2 was for example delivered from the second speaker on the left it was associated with a delay of 65ms, when it was delivered from the third speaker on the left with a delay of 130ms (65+65ms), and so on. In this condition, temporal cues could be used by subjects to infer spatial metric. Instead, space distances between the three sounds were inversely proportional to temporal intervals in the *opposite time* spatial bisection task (e.g. a shorter spatial distance between S1-S2 was associated with a longer temporal delay between the two sounds), making time informative but in the opposite direction with respect to space. Again, the exact temporal delay associated with each spatial position of S2 is reported in the upper horizontal axis of the psychometric function on the right in Figure 1. In this case for instance, when S2 was delivered from the second speaker on the left it was associated with a delay of 1435ms (1500-65ms), when it was delivered from the third speaker on the left with a delay of 1370ms (1435-65ms), and so on.

As regards the temporal bisection task performed as a control experiment, participants were asked to verbally report whether S2 was closer to S1 or to S3 in the time domain. Differently, to spatial bisection tasks, S2 occurred randomly from 0ms to 1500ms after S1 but it was always delivered from 0° in space, by the central speaker. Hence, exclusively temporal cues could be used to perform this task. As for S2 position in the spatial bisection tasks, the timing of S2 in the temporal bisection task was determined by the QUEST adaptive algorithm (Watson & Pelli, 1983).

All sighted subjects were blindfolded before entering the experimental room so that the exact location and layout of speakers could not be seen. Before testing, participants were warned to maintain a stable head position straight ahead. A short training session with feedbacks was conducted to make

participants familiar with the task and to be sure they understood it correctly. They were informed from the beginning that the first sound was always produced by a speaker placed on their left, whereas the last sound by a speaker on their right (as in original work, Gori et al., 2014). No feedbacks were given during experimental sessions.

Data analysis

For each task, we calculated the proportion of trials where the second sound was perceived as closer to the third sound and data were fitted by cumulative Gaussian functions. Following standard psychophysical procedure (Kingdom & Prins, 2010), PSE and threshold estimates were obtained from the mean and standard deviation of the best fitting function, and standard errors for the bisection PSE and threshold estimates were calculated by bootstrapping (Efron & Tibshirani, 1993). Analyses were subsequently conducted using R (R Core Team, 2017).

Some blind participants based their answers on temporal features in the *opposite time* spatial bisection task (i.e. when time intervals were incoherent with respect to space distances), exhibiting inverted psychometric functions. These corresponded to thresholds assuming negative values, with thresholds closer to 0 meaning good precision but in the temporal domain. In order to include these results together with those of blind individuals who performed the *opposite time* task without inverting the psychometric function, we applied a conversion to negative thresholds. Given thresholds (t) for the opposite time bisection task, negative values t_{neg} were converted to $t'_{neg} = t_{neg} - \min(t) + \max(t)$. This transformation allowed us to treat thresholds as a continuum, ranging from low thresholds representing good precision in the spatial domain to high thresholds representing poor spatial performance but good precision in the temporal domain.

To investigate spatial bisection precision, statistical comparisons between thresholds were performed with an omnibus two-way ANOVA, considering group (sighted, blind) as a between-subjects factor, and task (*equal*, *coherent*, *opposite*) as a within-subjects factor. For each group, a follow-up one-way ANOVA was carried out with the task (*equal*, *coherent*, *opposite*) as a within-subjects factor. As regards the temporal bisection task, thresholds were analyzed with a one-way ANOVA with group (sighted, blind) as a between-subjects factor. Post-hoc comparisons were conducted with two-tailed t-tests, with probabilities treated as significant when lower than 0.05 after Bonferroni correction.

Supplemental References

Efron, B., and Tibshirani, R. J. (1993). An Introduction to the Bootstrap (New York, NY: Chapman & Hall).

Gori, M., Sandini, G., Martinoli, C., and Burr, D. C. (2014). Impairment of auditory spatial localization in congenitally blind human subjects. *Brain* 137(1), 288-293.

Kingdom, F., and Prins, N. (2010). Psychophysics: a practical introduction (London, UK: Academic Press London).

Watson, A. B., and Pelli, D. G. (1983). QUEST: a Bayesian adaptive psychometric method. *Percept Psychophys* 33(2), 113-120.
